# Combining Ability of Different Agronomic Traits and Yield Components in Hybrid Barley

**DOI:** 10.1371/journal.pone.0126828

**Published:** 2015-06-10

**Authors:** Xinzhong Zhang, Liangjie Lv, Chao Lv, Baojian Guo, Rugen Xu

**Affiliations:** 1 Jiangsu Key Laboratory of Crop Genetics and Physiology, Yangzhou University, Yangzhou, China; 2 Co-Innovation Center for Modern Production Technology of Grain Crops, Yangzhou University, Yangzhou, China; 3 Key Laboratory of Plant Functional Genomics of the Ministry of Education, Yangzhou University, Yangzhou, China; 4 Institute of Barley Research, Yangzhou University, Yangzhou, China; University of Tasmania, AUSTRALIA

## Abstract

Selection of parents based on their combining ability is an effective approach in hybrid breeding. In this study, eight maintainer lines and nine restorer lines were used to obtain 72 crosses for analyzing the general combining ability (GCA) and special combining ability (SCA) for seven agronomic and yield characters including plant height (PH), spike length excluding awns (SL), inter-node length (IL), spikes per plant (SP), thousand kernel weight (TKW), kernel weight per plant (KWP) and dry matter weight per plant (DWP). The results showed that GCA was significantly different among parents and SCA was also significantly different among crosses. The performance of hybrid was significantly correlated with the sum of female and male GCA (TGCA), SCA and heterosis. Hu1154 A, Mian684 A, 86F098 A, 8036 R and 8041 R were excellent parents with greater general combining ability. Five crosses, Hu1154 A×8032 R, Humai10 A×8040 R, Mian684 A×8037 R, Mian684 A×8041 R and 86F098 A×8037 R, showed superior heterosis for most characters.

## Introduction

Barley (*Hordeum vulgare L*.) ranks the fourth in terms of planting area and total production among all cereal crops in the world [1]. It has been widely used as a health food, animal feed and fermentable material for the beer industry. Great progress has been made on barley heterosis studies [2] with the development of barley three-line breeding system based on cytoplasmic male sterility (CMS), maintainer and restorer lines [3]. The first commercial hybrid variety ‘Colossus’ was released in the UK in 2002 [4]. Since then, Syngenta *Ltd* released more than ten six-row winter hybrid barley varieties based on the CMS system. Over 200,000 ha of hybrid barley varieties were sown in Europe [4].

Heterosis exists widely in barley hybrids but varies greatly among crosses [2]. The mid-parent heterosis of barley grain yield ranged from 0.7 to 19.9% among different hybrids with an average value of 11.3%, while better-parent heterosis ranged from -1.7 to 18.3% with a slightly lower value of 9.2% [5]. Hence, selection of hybrids with superior heterosis from a large number of crosses is cost-effective in breeding programs. One of the key issues for the successful use of hybrid barley is to identify parents that have a high combining ability for producing hybrids with greater heterosis.

Evaluation of all possible crosses are time-consuming and laboursome in breeding programs. Certain lines have the ability to combine well with other lines, suggesting that these lines have good GCA. When an inbred combines well only in certain crosses, that means that it has good SCA [6]. Combining ability is effective for the selection of excellent parents in early generations [7]. GCA provides a simple approach to predict additive effects contributing to heterosis [8] and SCA also plays an important role on heterosis [9]. Combining ability has been successfully used to identify superior combinations in rice [10,11], maize [12,13,14] and wheat [15,16]. In barley, combining ability has been reported for various traits, including spike traits [17], flour pasting properties [18], salinity tolerance [19], and waterlogging tolerance [20]. Parents having high GCA values could be used to produce improved lines in hybridization programs and better hybrids can be produced in combination with high SCA values [17]. A moderate and significant correlation between mid-parent and hybrid performance and slightly lower correlation between the sum of GCA effects and performance of the hybrid itself were found when using 124 six-rowed winter barley hybrids based on CMS × restorer to examine the potential in predicting the hybrid performance of grain yield based on mid-parent values or GCA effects [5].

The objectives of this study were to: 1) study combining ability of different agronomic traits and yield components; and 2) study the relationships between heterosis and combining ability thus to provide a theoretical basis for parent selection in the use of hybrid varieties.

## Materials and Methods

### Materials and field experiment

Eight CMS lines (A) and nine restorer lines (R) (Table 1) were used to make 72 hybrids. All the lines were sourced from the Barley Research Institution of Yangzhou University.

**Table 1:** The hybrid barley parents and their row type in this experiment.

No.	Male sterile line	Row type	No.	Restoration line	Row type
A_1_	Sunong5078A	Two row	R_1_	8032R	Six row
A_2_	Hu1154A	Two row	R_2_	8033R	Six row
A_3_	Humai10A	Two row	R_3_	8034R	Two row
A_4_	89-0915A	Two row	R_4_	8035R	Two row
A_5_	Sunong5266A	Two row	R_5_	8036R	Two row
A_6_	Mian684A	Six row	R_6_	8037R	Two row
A_7_	84-161A	Two row	R_7_	8040R	Six row
A_8_	86F098A	Six row	R_8_	8041R	Six row
			R_9_	8042R	Two row

The experiment was performed at the Experimental Farm of Yangzhou University (119.4°E, 332.3°N) in the 2011 growing season. All 72 possible hybrids were produced by manual pollination. Forty seeds of each parent and hybrid were sown in a 5-row plot with a 1.2 m row-length and 20 cm between rows. The experiment was arranged in three replicates. For all trials, the fertilizer used included: 150 kg/ha before sown, 75 kg/ha at seedling stage and 75 kg/ha used at elongation stage. Aphids were sprayed at both seedling and flowering stages. Pinoxaden was applied to control weeds before winter. At maturity, eight plants were randomly taken from the middle line for the measurements of seven traitsplant height (PH), spike length excluding awns (SL), inter-node length (IL), spikes per plant (SP), thousand kernel weight (TKW), kernel weight per plant (KWP) and dry matter weight per plant (DWP).

### Statistical analysis

Based on the average value of eight plants, mid-parent (MP) and over-better-parent (OBP) heterosis for all traits were calculated by the formulae: MP heterosis = F_1_—[(P_1_ + P_2_) / 2] and OBP heterosis = F_1_—P_b_, where P_b_ stands for performance of the better parent [21,22]. General combining ability and special combining ability were calculated by the formulae:$g_{i={\bar{y}}_{i.-}\bar{y}},\;g_{j={\bar{y}}_{.j-}\bar{y}}$,${\text{s}}_{ij={\bar{y}}_{ij}-{\bar{y}}_{i.}-{\bar{y}}_{.j}+\bar{y}}$, where g_i_ and g_j_ stand for GCA of parent, s_ij_ stands for SCA of cross, $\bar{y}$_i._,$\bar{y}$._j_, $\bar{y}$_ij_ and $\bar{y}$ stand for the mean of crosses with same parent Pi, the mean of crosses with same parent Pj, the mean of crosses Pi/Pj, the mean of all crosses, respectively [23]. Descriptive statistics, ANOVA and correlation analysis were implemented using software Matlab 2010.

## Results

### Heterosis of different crosses

**Fig 1:**
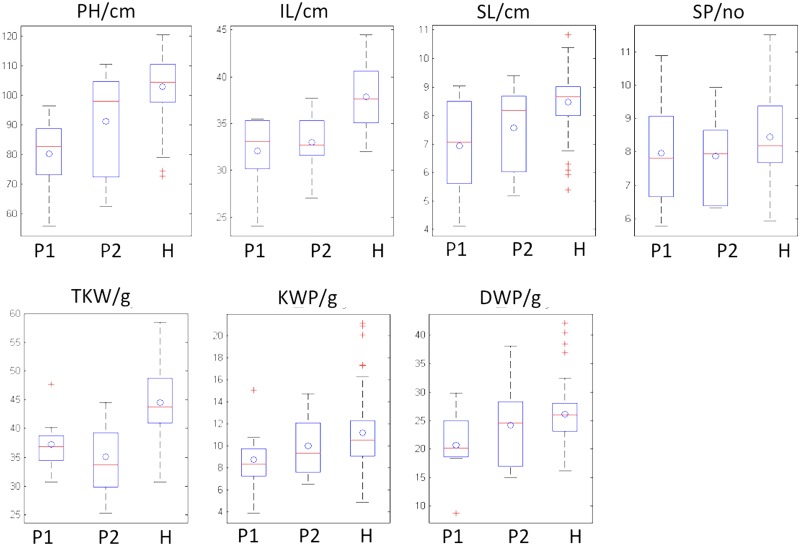
Box plots for performances of parents and crosses for seven measured traits. P1: female parents; P2: male parents; H: hybrids. The circle ‘o’ stands for mean value. The plus sign ‘+’ stands for outliers. The upper and lower lines outside the box stand for max and min adjacent value, respectively. The line inside the box stands for median value. The upper and lower hinge of the box stand for 75% and 25% percentile, respectively.

Heterosis was found in all crosses, for all measured barley traits (Fig 1). Significant differences were found among crosses for both MP and OBP heterosis. The degrees of variation for MP heterosis and OBP heterosis varied greatly among traits (Table 2) with the coefficient of variation ranging from 66.29 in SL to 223.34 in SP for MP heterosis and from 39.89 in PH to 3284.30 in KWP for OBP heterosis. The percentage of hybrids with significant heterosis ranged from 15.3% in SP to 97.2% in PH for MP heterosis and from 0% in PH to 72.2% in TKW for OBP heterosis.

**Table 2:** Performance of different characters of hybrid barleys.

Traits	Mid-parents heterosis				Over-better-parent heterosis			
	Mean	Range	*CV*	Significant percentage	Mean	Range	*CV*	Significant percentage
PH/cm	17.22	2.09~34.00	42.87	97.2%	27.36	8.03~57.93	39.89	0%
IL/cm	5.29	-0.20~12.64	56.43	81.9%	3.29	-2.89~9.88	92.03	68.1%
SL/cm	1.21	-0.54~2.83	66.29	76.4%	0.28	-1.54~2.46	277.56	20.8%
SP/no	0.53	-1.87~3.17	223.34	15.3%	-0.41	-2.97~2.39	324.69	5.6%
TKW/g	8.31	-1.50~20.77	69.59	73.6%	4.90	-9.04~18.16	125.95	72.2%
KWP/g	1.78	-3.08~9.75	130.96	16.7%	0.09	-7.21~7.29	3284.30	9.7%
DWP/g	3.68	-6.96~16.41	130.32	33.3%	-0.54	-15.92~9.75	1110.07	8.33%

PH: plant height; SL: spike length excluding awns; IL: inter-node length; SP: spikes per plant; TKW: thousand kernel weight; KWP: kernel weight per plant; DWP: dry matter weight per plant.

### Combining ability

ANOVA of combining ability showed the variances of GCA were significant for all traits in both female and male parents (Table 3). The variances of SCA were significant only for traits PH, IL, SL and TKW. The ratio of TGCA/SCA ranged from 6.24 in IL to 18.87 in SP, indicating that additive effects played a more important role than non-additive effects for all traits.

**Table 3:** ANOVA of combining ability for different traits (*F value*).

	PH	IL	SL	SP	TKW	KWP	DWP
Female GCA	27.20**	14.69**	46.10**	9.38**	29.52**	16.17**	11.62**
Male GCA	81.72**	32.95**	47.60**	6.09**	28.41**	4.05**	6.57**
SCA	7.13**	7.64**	5.62**	0.82	5.37**	1.18	1.30
GCA/SCA	15.28	6.24	16.67	18.87	10.79	17.14	13.99

*, ** significant at p < 0.05, 0.01 respectively.

GCA: general combining ability; SCA: special combining ability

The GCA of different varieties varied significantly among traits (Table 4). For example, the GCA of Sunong5078A was negative for IL, SL, KWP and DWP but positive for SP. Except for PH (higher PH leading to a greater chance of lodging), high positive GCAs are preferred for all the other six traits. Four parents, 89-0915A, 8032R, 8040R and 8042R, were better for PH. Seven parents, Hu1154A, Suning5266A, 86F098A, 8033R, 8035R, 8036R and 8041R, were better for IL. Eight parents, Hu1154A, Humai10A, Sunong5266A, 86F098A, 8034R, 8035R, 8037R and 8041R were better for SL. Five parents, Sunong 5078A, Hu1154A, Humai10A, 8036R and 8037R, were better for SP. Six parents, Hu1154A, 89-0915A, Sunong5266A, 8033R, 8036R and 8041R, were better for TKW. Three parent, Mian684A, 86F098A and 8041R, were better for KWP and DWP. In terms of the performance of all traits, Hu1154A, Mian684, 86F098A, 8036R and 8041R had a preferred GCA for breeding programs.

**Table 4:** General combining ability for agronomic and yield characters.

Parents	PH	IL	SL	SP	TKW	KWP	DWP
Sunong5078A	0.47	-2.48**	-0.54**	1.13**	-0.98	-1.27*	-1.72*
Hu1154A	0.91	1.34**	0.82**	1.04**	4.64**	-0.27	-0.57
Humai10A	4.96**	-0.06	0.56**	0.88**	0.81	0.69	1.57
89-0915A	-9.07**	-0.89**	-0.69**	0.15	4.22**	-3.28**	-3.70**
Sunong5266A	1.78*	1.05**	0.40**	-0.96**	1.28*	-0.57	-0.66
Mian684A	-0.75	0.23	-0.75**	-1.06**	-2.66**	2.24**	2.02*
84-161A	-1.42	0.21	0.02	-0.48	-2.88**	-2.04**	-2.79**
86F098A	3.11**	0.61*	0.19*	-0.69*	-4.44**	4.48**	5.84**
*PLSD*_*0*.*05*_	1.50	0.59	0.16	0.56	1.14	1.14	1.68
*PLSD*_*0*.*01*_	1.99	0.78	0.22	0.74	1.52	1.52	2.23
8032R	-10.50**	-0.63*	-1.15**	-0.44	-1.91**	1.02	0.47
8033R	6.21**	2.72**	0.09	-0.31	2.34**	0.58	1.76
8034R	2.38**	-2.21**	0.97**	-0.63*	0.00	-1.81**	-3.33**
8035R	4.22**	1.44**	0.19*	0.11	-1.29*	-0.30	-2.08*
8036R	3.38**	1.15**	-0.07	1.16**	5.35**	-0.45	0.65
8037R	1.47	-2.76**	0.47**	1.24**	-2.24**	-1.37*	-1.26
8040R	-6.45**	-0.59	-0.43**	-1.02**	-1.38*	0.05	-1.01
8041R	10.78**	2.07**	0.52**	-0.43	4.54**	2.60**	5.11**
8042R	-11.50**	-1.19**	-0.60**	0.31	-5.41**	-0.32	-0.31
*PLSD*_*0*.*05*_	1.60	0.63	0.18	0.60	1.22	1.22	1.79
*PLSD*_*0*.*01*_	2.13	0.84	0.23	0.79	1.62	1.62	2.38

*, ** significant at p < 0.05, 0.001 respectively.

See Table 2 for trait abbreviations

Forty eight crosses had a preferred SCA for at least one trait (S1 Table). Several crosses showed preferred SCA for PH, IL, SL, SP, TKW and DKW, respectively (Table 5). Hu1154A, Mian684A, 86F098A, 8032R, 8033R and 8040R had a preferred SCA in more crosses for different traits. Five crosses, Hu1154A×8032R, Humai10A×8040R, Mian684A×8037R, Mian684A×8041R and 86F098A×8037R, had a preferred SCA for at least three traits. The two parents of those five crosses all had different row types.

**Table 5:** Crosses with beneficial special combining ability for the seven traits.

PH	IL	SL	SP	TKW	KWP	DWP
A1×R2	A1×R1	A1×R1	A1×R4	A1×R9	A6×R7	A2×R1
A1×R5	A1×R7	A2×R1	A2×R1	A2×R1	A6×R8	A6×R8
A1×R9	A2×R1	A2×R3		A2×R2	A8×R2	A8×R9
A2×R8	A2×R2	A2×R4		A2×R7		
A3×R4	A2×R7	A3×R7		A2×R8		
A3×R6	A3×R1	A4×R2		A3×R2		
A4×R1	A3×R7	A5×R1		A3×R7		
A4×R5	A4×R2	A5×R9		A4×R9		
A4×R7	A4×R8	A6×R2		A6×R3		
A6×R1	A4×R9	A6×R6		A6×R5		
A6×R7	A5×R2	A6×R8		A6×R6		
A7×R3	A6×R4	A7×R9		A7×R7		
A7×R6	A6×R5	A8×R4		A8×R3		
A8×R1	A6×R6	A8×R5		A8×R6		
A8×R2	A7×R1	A8×R6				
A8×R8	A7×R2					
	A8×R4					
	A8×R5					
	A8×R6					
	A8×R9					

See Table 2 for trait abbreviations.

### Correlation between hybrid performance, heterosis and combining ability

Significant correlation was found between combining ability and heterosis (Table 6). For all traits, both MP and OBP heterosis were significantly correlated with SCA. For the yield components SP, TKW and KWP, heterosis was also significantly correlated to total GCA (the sum of female GCA and male GCA, TGCA). The performance of hybrids had a significant correlation with heterosis and combining ability (Table 7). The correlation coefficients between hybrid performance with heterosis were slightly lower than those between hybrid performance with combining ability. The correlation coefficients between hybrid performance with TGCA were much higher than those between hybrids performance with SCA for all traits except IL.

**Table 6:** Correlation between heterosis and combining ability.

Traits	Heterosis	FGCA	MGCA	TGCA	SCA
PH	MP	-0.19	-0.08	-0.17	0.78**
	OBP	-0.40**	0.11	-0.09	0.68**
IL	MP	0.06	0.18	0.18	0.78**
	OBP	0.18	0.18	0.25*	0.79**
SL	MP	-0.19	-0.04	-0.15	0.70**
	OBP	0.09	-0.08	0.00	0.36**
SP	MP	0.26*	0.35**	0.42**	0.60**
	OBP	0.10	0.34**	0.30*	0.53**
TKW	MP	0.36**	0.31**	0.47**	0.65**
	OBP	0.30*	0.29*	0.42**	0.57**
KWP	MP	0.43**	0.08	0.41**	0.76**
	OBP	0.36**	-0.11	0.27*	0.71**
DWP	MP	0.15	0.12	0.19	0.57**
	OBP	0.21	-0.08	0.11	0.49**

*, ** significant at p < 0.05, 0.001 respectively.

See Table 2 for trait abbreviations.

FGCA: female general combining ability; MGCA: male general combining ability; TGCA: total general combining ability, TGCA = FGCA+MGCA; SCA: special combining ability.

MP: mid-parents heterosis; OBP: over-better-parent heterosis.

**Table 7:** Correlation between the performance of hybrids and combining ability.

	MP	OBP	FGCA	MGCA	TGCA	SCA
PH	0.30**	0.31**	0.39**	0.73**	0.82**	0.57**
IL	0.70**	0.75**	0.36**	0.58**	0.68**	0.73**
SL	0.26*	0.20	0.56**	0.61**	0.83**	0.56**
SP	0.68**	0.54**	0.64**	0.55**	0.85**	0.53**
TKW	0.78**	0.69**	0.53**	0.56**	0.77**	0.64**
KWP	0.77**	0.62**	0.73**	0.39**	0.83**	0.56**
DWP	0.49**	0.38**	0.63**	0.50**	0.81**	0.59**

*, ** significant at p < 0.05, 0.001 respectively.

See Table 2 for trait abbreviations.

FGCA: female general combining ability; MGCA: male general combining ability; TGCA: total general combining ability, TGCA = FGCA+MGCA; SCA: special combining ability.

MP: mid-parents heterosis; OBP: over-better-parent heterosis.

## Discussion

Barley hybrid breeding has been remarkably successful in past decades. However, due to the lack of high yielding heterotic patterns and the lower selection gain for hybrid compared to traditional breeding, only a few hybrid barley varieties have been released [4]. Selection of superior hybrids based on plant performance is more practical but more time-consuming. Combining ability is considered to be a useful indirect criterion for parent selection. However, only one study on combining ability of hybrid barley grain yield has been reported to our knowledge [5]. In fact, both agronomic and yield traits are important in hybrid barley breeding programs [2]. In this study, seven important traits PH, SL, IL, SP, TKW, KWP and DWP were investigated. GCA for all traits was significant while SCA was significant only for traits PH, IL, SL and TKW. The ratio of TGCA/SCA revealed that additive effects were the main effect on barley traits, which was the same in previous studies on barley [17] and maize [12]. Therefore, it is necessary to select parents with high general GCA, which would benefit the offspring. In this study, Hu1154A, Mian684, 86F098A, 8036R and 8041R were excellent parents with greater general combining ability.

Prediction of hybrid performance and heterosis is important in hybrid barley breeding. Heterosis and combining ability are two main indexes for hybrid performance. In this study, heterosis was significantly correlated with SCA for all agronomic and yield characters, indicating non-additive effects were the main effect for heterosis. However, mid-parent heterosis ignores non-additive effect, an important criterion in hybrid performance [24]. In contrast, the correlation coefficients between hybrids performance and TGCA were higher than those between hybrids performance and heterosis, which is different from the study by Mühleisen et al. [5]. In this study, we also found that the hybrid performance had a higher correlation with TGCA than with SCA. Hence, GCA is much more important than SCA in hybrid barley breeding. Parents with high GCAs can be crossed to select superior combinations.

## Conclusion

Using combining ability to select parents is an effective approach in hybrid breeding. The current study presented the first report on the combining ability for seven main agronomic and yield characters. GCA was significantly different among parents and SCA was also significantly different among crosses. The hybrids from two parents with high GCA always showed better hybrid performance even though SCAs were low. Thus the selection of parents should mainly be based on their GCAs.

## Supporting Information

S1 Table Special combining ability for agronomic and yield characters. (DOCX)
