# Supplementary material for: Combining Ability of Different Agronomic Traits and Yield Components in Hybrid Barley
Source: PLoS One. 2015 Jun 10;10(6):e0126828. doi: 10.1371/journal.pone.0126828 (PMC4465181; doi:10.1371/journal.pone.0126828)
Supplement: S1 Table — (DOCX) [file pone.0126828.s001.docx]

Table S1 Special combining ability for agronomic traits and yield components

| Crosses | PH | IL | SL | SP | TKW | KWP | DWP |
| --- | --- | --- | --- | --- | --- | --- | --- |
| Sunong5078×8032R | 5.78** | 1.65* | 0.52* | -0.37 | 2.66 | 0.05 | 2.1 |
| Sunong5078×8033R | -4.76* | -3.32** | -0.15 | -0.13 | -1 | -1.13 | -4.11 |
| Sunong5078×8034R | 3.05 | 1.26 | -0.16 | -0.01 | -0.8 | 0.88 | 1.26 |
| Sunong5078×8035R | -3.7 | -0.53 | -0.66** | 1.57* | -0.28 | 0.8 | 3.54 |
| Sunong5078×8036R | -6.25** | -1.63 | 0.16 | 0.56 | -1.69 | 1.07 | -0.24 |
| Sunong5078×8037R | -0.77 | -0.51 | -0.14 | 0.09 | -5.70** | 0.47 | 0.54 |
| Sunong5078×8040R | 5.76** | 2.58** | 0.41 | 0.61 | -0.59 | -0.83 | 0.25 |
| Sunong5078×8041R | 5.30* | 1.25 | -0.14 | -1.24 | 2.65 | -0.35 | -1.82 |
| Sunong5078×8042R | -4.42* | -0.76 | 0.17 | -1.08 | 4.75** | -0.95 | -1.51 |
| Hu1154×8032R | 4 | 2.68** | 0.51* | 2.11** | 3.43* | 1.14 | 4.81* |
| Hu1154×8033R | -3.35 | 1.82* | -0.97** | -0.36 | 4.38** | -1.8 | -1.34 |
| Hu1154×8034R | -0.04 | -1.33 | 0.57* | -0.75 | -6.45** | -0.15 | 0.12 |
| Hu1154×8035R | -0.21 | 0.55 | 0.75** | -1.45 | -4.38** | -0.44 | -2.22 |
| Hu1154×8036R | 1.63 | -1.44 | -0.55* | 0.84 | -1.44 | 1.88 | -0.05 |
| Hu1154×8037R | -2.42 | -0.73 | -0.16 | -1.4 | -3.79* | 0.68 | 0.13 |
| Hu1154×8040R | 0.17 | 1.76* | -0.07 | 1.13 | 4.36** | 1.36 | 3.28 |
| Hu1154×8041R | -4.21* | -0.03 | -0.28 | -0.23 | 4.70** | -1.77 | -2.89 |
| Hu1154×8042R | 4.42* | -3.29** | 0.2 | 0.12 | -0.81 | -0.9 | -1.84 |
| Humai10×8032R | 9.74** | 4.03** | 0.27 | -0.62 | 2.63 | 0.71 | 2.22 |
| Humai10×8033R | -1.78 | 0.49 | -0.47* | -0.31 | 3.53* | -1.15 | -1.24 |
| Humai10×8034R | -3.78 | -0.62 | 0.01 | 1.01 | -3.16 | 1.74 | 3.21 |
| Humai10×8035R | -5.02** | -3.03** | -0.33 | 0.16 | -0.54 | 0.2 | -1.2 |
| Humai10×8036R | -2.22 | -1.85* | 0.13 | 0.22 | -4.16* | -0.57 | 0.75 |
| Humai10×8037R | -7.48** | -2.51** | -0.39 | 0.53 | -4.38** | 1.19 | 0 |
| Humai10×8040R | 11.32** | 4.29** | 0.97** | -0.25 | 8.04** | 0.34 | 0.51 |
| Humai10×8041R | 1.64 | 0.83 | -0.27 | -0.47 | 1.84 | -1.25 | -2.23 |
| Humai10×8042R | -2.43 | -1.63 | 0.08 | -0.27 | -3.81* | -1.2 | -2.02 |
| 89-0915×8032R | -8.86** | -4.30** | 0.15 | -0.01 | -1.82 | -3.01 | -3.48 |
| 89-0915×8033R | 10.95** | 3.38** | 0.67** | 0.32 | 0.35 | 0.73 | 1.92 |
| 89-0915×8034R | 3.38 | 1.24 | 0.08 | -0.1 | 0.49 | 1.37 | 2.92 |
| 89-0915×8035R | 0.8 | -2.05* | -0.05 | -0.21 | 1.53 | 1.26 | 1.18 |
| 89-0915×8036R | -5.35* | 0.18 | -0.15 | 0.12 | -0.59 | 1.15 | 1.21 |
| 89-0915×8037R | 0.87 | 0.75 | -0.38 | -0.43 | -3.31* | 1.62 | 0.6 |
| 89-0915×8040R | -14.73** | -4.00** | -0.41 | -0.74 | -2.15 | -3.07 | -5.18* |
| 89-0915×8041R | 9.15** | 2.26** | 0.03 | 0.07 | 0.38 | -2.53 | -0.77 |
| 89-0915×8042R | 3.79 | 2.54** | 0.07 | 0.99 | 5.12** | 2.48 | 1.59 |
| Sunong5266×8032R | 3.45 | 0.93 | 0.94** | 0.21 | -1.98 | 0.54 | -0.49 |
| Sunong5266×8033R | 1.36 | 1.96* | 0.06 | 0.91 | -2.5 | -0.4 | -1.06 |
| Sunong5266×8034R | -3.3 | -1.97* | -0.57* | 0.25 | 1.83 | 0.56 | 0.6 |
| Sunong5266×8035R | -0.84 | -0.51 | -0.26 | -0.21 | 1.94 | -0.89 | -0.69 |
| Sunong5266×8036R | 2.21 | 1.56 | -0.68** | -1.81* | 1.34 | 0.49 | 0.42 |
| Sunong5266×8037R | -3.05 | -1.70* | -0.16 | 1.4 | 1.87 | 1.34 | 3.72 |
| Sunong5266×8040R | 0.22 | 0.59 | 0 | -0.57 | -5.73** | -1.65 | -1.52 |
| Sunong5266×8041R | -0.16 | -0.44 | 0.19 | 0.6 | 2.68 | 1.24 | 0.71 |
| Sunong5266×8042R | 0.12 | -0.43 | 0.48* | -0.77 | 0.55 | -1.25 | -1.69 |
| Mian684×8032R | -12.76** | -4.58** | -1.19** | -0.76 | -3.09 | 0.6 | -6.31** |
| Mian684×8033R | 0.7 | -1.48 | 1.05** | -0.81 | -6.01** | 2.1 | 2.48 |
| Mian684×8034R | 3.96 | 1.6 | 0.12 | 0.9 | 3.84* | -2.58 | -2.32 |
| Mian684×8035R | 7.50** | 3.85** | 0.35 | 0.2 | 3.1 | -1.81 | 0.63 |
| Mian684×8036R | 7.09** | 1.80* | 0.36 | -0.73 | 6.70** | -3.69* | -2.44 |
| Mian684×8037R | 7.80** | 2.07* | 0.66** | 0.58 | 10.88** | -2.54 | 1.27 |
| Mian684×8040R | -12.16** | -2.70** | -1.19** | -0.14 | -4.90** | 3.80* | 1.85 |
| Mian684×8041R | -1.01 | -1.53 | 0.68** | 0.27 | -4.90** | 4.87** | 8.67** |
| Mian684×8042R | -1.11 | 0.97 | -0.83** | 0.48 | -5.62** | -0.76 | -3.83 |
| 84-161×8032R | 9.01** | 3.33** | 0.4 | -0.75 | -0.56 | 0.38 | 1.59 |
| 84-161×8033R | 2.87 | 2.09* | -0.4 | 0.16 | 2.71 | -2.18 | 0.16 |
| 84-161×8034R | -5.19* | -0.59 | -0.44 | -0.95 | 0.73 | 0.52 | -3.1 |
| 84-161×8035R | -2.59 | -1.6 | -0.33 | 0.1 | 0.57 | 0.88 | -0.5 |
| 84-161×8036R | -2.47 | -3.47** | 0.2 | 0.36 | -1.35 | 2.35 | 4.24 |
| 84-161×8037R | -6.16** | -0.13 | -0.71** | 0.45 | -5.43** | 0.41 | -1.53 |
| 84-161×8040R | 3.85 | 0.36 | 0.13 | -0.21 | 3.61* | 0.07 | 1.44 |
| 84-161×8041R | -1.51 | 0.21 | 0.26 | 0.27 | -2.83 | -3.05 | -4.89* |
| 84-161×8042R | 2.21 | -0.2 | 0.89** | 0.56 | 2.55 | 0.63 | 2.59 |
| 86F098×8032R | -10.35** | -3.75** | -1.59** | 0.18 | -1.27 | -0.41 | -0.45 |
| 86F098×8033R | -5.99** | -4.95** | 0.23 | 0.23 | -1.46 | 3.82* | 3.19 |
| 86F098×8034R | 1.92 | 0.4 | 0.4 | -0.36 | 3.50* | -2.35 | -2.68 |
| 86F098×8035R | 4.07 | 3.32** | 0.53* | -0.15 | -1.95 | 0.01 | -0.75 |
| 86F098×8036R | 5.36* | 4.85** | 0.53* | 0.44 | 1.2 | -2.67 | -3.89 |
| 86F098×8037R | 11.21** | 2.77** | 1.28** | -1.2 | 9.87** | -3.18 | -4.73* |
| 86F098×8040R | 5.58** | -2.89** | 0.15 | 0.17 | -2.63 | -0.02 | -0.62 |
| 86F098×8041R | -9.22** | -2.56** | -0.47* | 0.72 | -4.52** | 2.84 | 3.22 |
| 86F098×8042R | -2.58 | 2.79** | -1.07** | -0.04 | -2.74 | 1.97 | 6.71** |
| *PLSD*_0.05_ | 4.19 | 1.65 | 0.46 | 1.56 | 3.2 | 3.19 | 4.69 |
| *PLSD*_0.01_ | 5.51 | 2.17 | 0.61 | 2.05 | 4.2 | 4.2 | 6.17 |

*, ** significant at p < 0.05, 0.001 respectively.

See Table 2 for abbreviation of trait.
